# Liver Transplantation for Cancer—Current Challenges and Emerging Solutions

**DOI:** 10.3390/jcm14155365

**Published:** 2025-07-29

**Authors:** Steven M. Elzein, Elizabeth W. Brombosz, Sudha Kodali

**Affiliations:** 1 Department of Surgery, Houston Methodist Hospital, 6550 Fannin Street Suite 1661, Houston, TX 77030, USA; smelzein@houstonmethodist.org (S.M.E.);; 2 Sherrie and Alan Conover Center for Liver Disease and Transplantation, J.C. Walter Jr. Transplant Center, Houston Methodist Hospital, Houston, TX 77030, USA; 3 Department of Medicine, Houston Methodist Hospital, Houston, TX 77030, USA; 4 Department of Medicine, Weill Cornell Medical College, New York, NY 10065, USA

**Keywords:** liver transplantation, transplant oncology, hepatocellular carcinoma, cholangiocarcinoma, liver neoplasms

## Abstract

Liver transplantation (LT) for hepatic malignancies is becoming increasingly common, largely because it offers superior survival relative to other treatment approaches. LT is well-accepted for primary liver cancers such as hepatocellular carcinoma and perihilar cholangiocarcinoma and is being increasingly accepted for intrahepatic cholangiocarcinoma and metastases of colorectal cancer or neuroendocrine tumors to the liver. Over time, indications for transplant oncology have broadened, as has the acceptable disease burden for transplantation, particularly with the advent of new neoadjuvant therapies. Other current frontiers in the field include expanding the donor pool through living donors, extended criteria donors, machine perfusion and increasing access to LT for people from disadvantaged socioeconomic backgrounds. Expanding access to LT can offer renewed hope for long-term survival to patients with primary and secondary liver cancer.

## 1. Introduction

Liver transplantation (LT) has established itself as a life-saving intervention for patients suffering from end-stage liver disease. LT generally provides superior long-term survival than resection [1], and is frequently the treatment of choice in patients with non-resectable liver cancer. As the utilization of LT in the United States expands, experiencing a 52% increase in the past decade [2], patients with liver cancer also are increasingly being transplanted. Of the 9527 LTs performed in 2022, 10.9% were for a primary diagnosis of hepatocellular carcinoma (HCC), the fourth-most common indication overall [2]. Despite its limitations, LT for hepatocellular carcinoma (HCC) has become the mainstay of therapy for eligible recipients, with excellent five-year graft and patient survival rates greater than 75% [2].

In addition to HCC, other primary and secondary malignancies of the liver, including intrahepatic (iCCA) and hilar cholangiocarcinoma (hCCA), colorectal liver metastases (CRLM), and neuroendocrine tumors (NET) pose their own unique challenges to liver transplantation as a viable treatment modality. In addition to inherent tumor biology, which if aggressive precludes transplantation, obstacles to increased success for a wider range of malignancies include the lack of MELD exception points for some cancers (Table 1), surgical difficulties created by neoadjuvant therapy, long wait times, lack of prospective data, and relatively decreased overall survival, amongst others. Although several reports review the current landscape of liver transplant oncology [3,4,5,6], few highlight the challenges associated with successful liver transplantation for primary and secondary malignancy. This narrative review puts forth evidence for the current challenges in LT for primary and secondary cancers and discusses emerging solutions for LT in this cohort.

## 2. Challenges in Liver Transplantation for Primary Liver Cancer

### 2.1. Hepatocellular Carcinoma

Although initial attempts at LT involved primary malignancies [7], high post-transplant recurrence and mortality rates resulted in liver cancer being a contraindication for LT for many years. A major paradigm shift in transplant oncology came when Mazzaferro and colleagues published the post-LT outcomes of patients with unresectable HCC within Milan Criteria in 1996 [8]. These criteria defined an upper limit to the size and number of unresectable HCC lesions a patient could have and still expect low recurrence and high survival post-LT (Table 2). Since this seminal publication, there has been a gradual expansion in the accepted limits of disease burden that can be transplanted safely [9,10,11]. Many centers now use selection criteria that include both numeric tumor burden (size and number) and alpha fetoprotein (AFP) [12,13]. Candidate selection assessing tumor biology through response to neoadjuvant therapy and radiographic stability, regardless of tumor burden and AFP levels, has also shown promise [14].

LT for HCC has also benefitted greatly from the surge in new locoregional and systemic therapies for this disease. Neoadjuvant treatments can be used to downstage a patient’s disease to within criteria limits or as a bridging treatment to transplantation, allowing previously out-of-criteria patients to undergo LT with excellent results [21,22].

Radiation therapy (RT) advancements allow for targeted, safe delivery of radiation to HCC. Both retrospective and prospective studies have RT can lead excellent local tumor control and survival outcomes. Local control with doses of 30 to 90 Gy ranging from 71% to 100% with total doses between 30 and 90 Gy over 1 to 8 weeks [23,24]. Recent studies have shown that stereotactic body radiotherapy (SBRT) can be an effective downstaging therapy, but patients downstaged with SBRT may have a higher risk of post-transplant recurrence than those receiving SBRT as a bridging therapy [25], suggesting neoadjuvant SBRT may be best suited for bridging to transplant.

For patients with HCC and portal venous tumor thrombus (PVTT), RT has shown promising results, even in cases considered unfavorable. Multiple studies have reported response rates ranging from 37.5% to 100%, with median survival times between 3.8 and 10.7 months [26,27,28,29,30]. Retrospective data from MD Anderson Cancer Center (MDACC) further support these findings, indicating that patients with PVTT who received higher doses of RT experienced better local tumor control and longer survival compared to those treated with lower doses. These outcomes highlight the potential of RT as an effective treatment option for this challenging subset of HCC patients [31].

Liver resection and transplantation remain the only potentially curative treatments for HCC. However, only a small proportion of patients—approximately 10% to 30%—are eligible for these options. Preoperative, or neoadjuvant, therapy has been explored as a strategy to improve outcomes in hepatocellular carcinoma (HCC), particularly by aiming to shrink tumors and reduce micrometastatic disease before surgery. In a phase 2 open-label study, patients received four weeks of sorafenib prior to surgical resection. The results showed that 24% of resected tumors exhibited greater than 50% necrosis, and the study reported an objective response rate (ORR) of 32% based on modified RECIST (mRECIST) criteria. Although the study was small, it provided early evidence supporting the potential role of neoadjuvant therapy in improving surgical outcomes for HCC [32].

New immunotherapy drugs for HCC are being investigated as neoadjuvant treatment for LT [33], generally with a washout period to reduce the risk of graft rejection. These treatments are allowing the expansion of LT to more patients with greater tumor burden, ultimately extending the lives of many patients with HCC.

Early investigations into preoperative immunotherapy for resectable HCC showed encouraging results. In a pilot randomized trial evaluating perioperative treatment with nivolumab alone or in combination with ipilimumab, 5 out of 21 patients who underwent resection achieved a complete pathological response—2 in the nivolumab group and 3 in the combination group [34]. Several other regimens of combination chemoimmunotherapy have been approved in the last few years which have shown promising results, though there are no established systemic preoperative therapies for resectable HCC. Ongoing clinical trials involving tyrosine kinase inhibitors and immune checkpoint inhibitors are expected to provide further insight into the role of neoadjuvant therapy in this setting.

### 2.2. Adjuvant Chemotherapy

In the early 2000s, adjuvant therapy for HCC produced inconsistent outcomes. A recent large, phase 3, randomized trial using sorafenib as an adjuvant therapy showed no significant difference in median recurrence-free survival between the sorafenib and placebo groups (33.3 vs. 33.7 months), and patients receiving sorafenib experienced more adverse effects, including hand-foot syndrome and elevated liver enzymes [35]. At present, there is no convincing regimen that is considered standard of care for HCC in the post resection and transplant setting.

Hepatic artery infusion pump (HAIP) therapy is increasingly utilized in the treatment of intrahepatic tumors including HCC, cholangiocarcinoma and colorectal liver metastases [36]. The pump allows delivery of high concentrations of chemotherapy directly to the liver and has been shown to improve response rates compared to chemotherapy alone. A known complication is biliary sclerosis, which can occur in up to 5–22% of patients and currently lacks a standardized treatment approach and can make transplantation challenging [37]. In certain cases, LT has been explored not only as a therapeutic option for HAIP-induced cholangiopathy but also as a potential definitive oncologic treatment following HAIP as a bridging strategy. A single-center study by Hill et al. showed that LT is a feasible treatment option for end-stage liver disease after HAIP therapy [38]. Technical considerations include a more challenging dissection and an atypical arterial anastomosis.

### 2.3. Perihilar Cholangiocarcinoma

Cholangiocarcinoma (CCA) is an adenocarcinoma of the biliary tract and generally has a poorer prognosis than HCC. It is generally classified by its location—intrahepatic (within the liver parenchyma), perihilar (in the region of the liver hilum), or distal (the common bile duct distal to its junction with the cystic duct). While patients with distal CCA are not candidates for LT, transplantation is increasingly common for highly selected patients with pCCA and iCCA.

While LT for HCC became widely accepted after the acceptance of the Milan criteria, CCA remained a contraindication for many years. Transplant for CCA initially gained traction thanks to the excellent outcomes after LT for unresectable pCCA (Klatskin tumor) reported by clinicians from the Mayo Clinic [19,39,40]. In the Mayo protocol, patients with a pCCA lesion < 3 cm in diameter are eligible for LT following successful neoadjuvant chemoradiotherapy treatment [39]. Staging laparotomies were also performed to rule out extrahepatic disease. Patients treated under this protocol had 5-year survival rates of 82%. This success led the US United Network for Organ Sharing (UNOS) to allow MELD exception points for pCCA patients meeting these requirements [41]. Some more recent studies have reported lower survival rates [42,43,44,45,46], but pCCA continues to be a well-accepted indication for LT.

### 2.4. Intrahepatic Cholangiocarcinoma

Acceptance of LT for iCCA began following a report describing outcomes of patients found to have incidental iCCA on explant. Sapisochin and colleagues found patients with “very early” iCCA, which they defined as a single lesion ≤ 2 cm in diameter, and cirrhosis had a 5-year overall survival rate post-LT of 73% [47]. A follow-up study reported 65% 5-year survival in patients with “very early” iCCA [18]. Other studies have described 5-year overall survival rates of 46–69% in patients with cirrhosis and small iCCA lesions [48,49,50,51,52]. Conversely, Lunsford et al. found 83.3% 5-year survival in a cohort of patients selected for amenable biology, regardless of the size and number of tumors [53]. In this protocol, patients with liver-limited iCCA receive neoadjuvant systemic therapy (most frequently gemcitabine-cisplatin), followed by a 6-month observation period. Patients showing disease response or stability radiographically are eligible for LT. A follow-up study by the same group reported 5-year overall survival of 57% in an expanded cohort [54]. Similarly, a 2022 paper from Ito and colleagues found that post-LT survival was not linked to tumor size, but was affected by the type of neoadjuvant therapy received [55].

As a result of these advancements in LT for iCCA, the Organ Procurement and Transplantation Network (OPTN) announced that patients with unresectable iCCA and cirrhosis with lesions ≤ 3 cm are eligible for exception points as of February 2025 [41]. Patients must also have been treated with neoadjuvant locoregional or systemic therapy and must have radiographically stable disease for at least 6 months. It has been suggested that LT should be restricted to patients with unresectable iCCA in the background of cirrhosis, but this limitation is not universally agreed upon by all experts [56].

### 2.5. Rare Primary Tumors

Infrequently, LT has been used to treat rarer tumor types, such as mixed hepatocellular-cholangiocarcinoma (HCC-CCA) [57,58], hepatic epithelioid hemangioendothelioma (HEHE) [59,60], hepatic adenoma [61], and hepatoblastoma (generally in pediatric patients) [62]. Patients with biopsy-proven, unresectable HEHE are eligible for UNOS MELD exception points [41]. To receive MELD exception points for adenoma, patients must have glycogen storage disease and an unresectable, progressive, β-catenin-positive lesion that is unresponsive to other treatments. Patients with HCC-CCA meeting the iCCA criteria may also receive MELD exception points. However, most patients with mixed HCC-CCA have poor overall and recurrence-free survival after LT, with survival rates much lower than patients with comparable HCC [63]. PELD exception points can be awarded to patients under the age of 8 with unresectable hepatoblastoma.

## 3. Challenges in Liver Transplantation for Secondary Liver Cancer

### 3.1. Colorectal Liver Metastases

LT for metastatic colorectal cancer (CRC) is an uncommon procedure, accounting for a very small fraction of all liver transplants. However, following the excellent post-transplant survival outcomes reported from the SECA-I and SECA-II studies out of Norway [15,16,17], LT for CRLM is gaining more interest worldwide. According to a study analyzing data from the United Network for Organ Sharing (UNOS) database between December 2017 and March 2022, 46 patients underwent LT for colorectal liver metastases (CRLM) across 15 centers in the United States [64]. The TRANSMET trial, a multicenter, randomized controlled study has provided compelling evidence that liver transplantation combined with chemotherapy significantly improves survival in selected patients with permanently unresectable colorectal liver metastases, compared to chemotherapy alone [65]. Despite advancements and renewed interest in this approach, the overall number of liver transplants performed for metastatic colorectal cancer remains low primarily due to stringent patient eligibility, post-operative disease-free survival, and ethical considerations in resource allocation.

#### 3.1.1. Patient Eligibility

Several studies have emphasized the importance of strict selection criteria to improve outcomes in patients undergoing liver transplantation for colorectal cancer metastasis to the liver [15,16,66]. Previously studied criteria including liver-only metastasis [15,16,17,66,67,68], 6 week minimum of chemotherapy [17,66] or at least 10% [16] or sustained [68] response to chemotherapy, more than 1 [16] or 3 years from cancer diagnosis to transplantation [66], good performance status [15,66], node-negative disease [66], and excision of the primary tumor [17] may limit the number of eligible patients who may benefit from liver transplantation. The correct balance of strict selection criteria to improve outcomes with the need to provide treatment options to more patients has yet to be defined in this population. Given recent data suggesting improved post-LT outcomes in carefully selected patients with CRLM, there has been growing interest in a protocol-based approach to transplantation in this population [65].

#### 3.1.2. Recurrence-Free Survival

Post-transplant outcomes reflect pronounced challenges in establishing liver transplantation as a treatment modality for CRC metastasis. Although reported overall survival rates at one year post-transplant range from 83% to 100% [15,16,69], disease-free survival rates remain modest at 53% at 1 year and 35–38% at later time points [16,69]. Recently, the authors of the TRANSMET study showed that 5-year overall survival in patients who receiving LT and chemotherapy was 3% compared to 9% in patients who received chemotherapy alone in the per-protocol analysis. In the intention-to-treat population, overall survival was 57% and 13%, respectively. Progression-free survival at 5 years was 20% in the LT group vs. 0% in the chemotherapy group, with 72% of transplant recipients experiencing recurrence, mostly in the lungs and lymph nodes. Of those with recurrence, 46% underwent successful surgical or ablative interventions. Around 42% of transplant patients had no evidence of disease at long-term follow-up, further strengthening the argument for transplant oncology and role of LT in patients with colorectal cancer with liver metastasis [65]. High recurrence rates—with times as short as 4 months and often with pulmonary involvement—further obfuscate transplantation as a durable treatment option [15,17].

#### 3.1.3. Ethical Considerations in Resource Allocation

Strict patient selection and high recurrence rates raise ethical implications for liver transplantation as a definitive treatment modality for metastatic colorectal cancer. Amidst uncertain criteria and outcomes, an intentional diversion of scarce donor organs is necessary to patients whose long-term success is largely unknown. Additionally, as it pertains to living donors, the potential for improved recipient survival must be weighed against the risks and morbidity of the donor operation. Lastly, full disclosure of the high likelihood of recurrent cancer in the recipient is an important ethical consideration to broach with the donor in the context of living donation.

### 3.2. Neuroendocrine Tumor Metastases

Liver transplantation for neuroendocrine tumor (NET) metastasis is relatively rare and represents approximately 0.2% to 0.3% of all liver transplants in the United States and Europe [70,71]. However, this strategy faces several key challenges, including patient selection, technical and surgical considerations, and poor long-term outcomes. For this reason, candidacy for LT has become increasingly selective, focusing primarily on patients with well-differentiated tumors, isolated hepatic metastasis, and stable disease.

#### 3.2.1. Patient Selection

Patient selection remains one of the key challenges in liver transplantation for NET metastasis, in part due to the scarcity of the indication. The absence of clearly defined patient selection criteria represents a major challenge to standardizing transplantation for NET. Along the same lines, overly restrictive criteria may deny potentially beneficial transplantation to some patients and further restrict this treatment modality [20]. Furthermore, the optimal way to incorporate criteria found to predict worse outcomes, including Ki-67 levels [72,73], tumor location in the pancreas [74,75], age > 45–50 years old [20,76], >50% tumor involvement of the liver [74], aberrant E-cadherin expression [73], and hepatomegaly [20] remains unclear.

#### 3.2.2. Technical and Surgical Considerations

The select few patients deemed suitable candidates for LT for NET metastasis face technical and surgical challenges as well. Perioperative risk is substantial, with Le Treut and colleagues reporting a 10% three-month postoperative mortality rate [20]. Furthermore, any amount of resection concurrent with LT was associated with poorer outcomes, suggesting that more extensive surgeries may increase risks without necessarily improving outcomes. Compounding these risks is the uncertainty regarding operative timing—Gedaly et al. found that longer wait times were associated with better outcomes [77], suggesting that the optimal timing may involve a period of disease stabilization rather than urgent operative intervention.

#### 3.2.3. Poor Long-Term Outcomes

Following transplant, poor survival and high recurrence rates plague the long-term success of LT for NET metastasis. After initial reports of lackluster 59% one-year post-transplant survival [78], recent advances have propelled reported one-year survival to as high as 89% [72,73,74], albeit in relatively small patient cohorts. Long-term survival remains elusive, with many studies reporting 5-year survival between 45 and 63% [20,74,75,76,77]. Furthermore, disease recurrence is of particular issue in this population, with reported recurrence rates near 57% five years post-transplant [72,74].

## 4. Emerging Solutions

### 4.1. Living Donor Liver Transplantation

In order to expand access to LT for primary and secondary liver cancer, the pool of available donor organs needs to grow accordingly. One such solution is living donor liver transplantation (LDLT). LDLT can reduce wait times relative to deceased donor LT (DDLT), lowering the risk of disease progression while on the waitlist that could render the patient transplant ineligible [79,80]. However, the benefits to the recipient must be balanced with the risks to the donor. In this setting, recipients must have a low risk of recurrence to balance the potential physical harm to the donor.

Many centers are employing minimally invasive approaches for donor hepatectomy, utilizing either laparoscopic and/or robotic surgical techniques. Minimally invasive hepatectomy has been shown to result in reduced intraoperative donor blood loss and shorter post-operative hospital stays [81]. Minimally invasive donor hepatectomy has also been associated with higher postoperative donor quality of life and improved satisfaction with their appearance after surgery [82]. Thus, minimally invasive donor hepatectomy may reduce the risks to the donor and improve the ethical balance between donor and recipient.

Most work in and benefit of living donor transplant oncology has been shown in patients with HCC. Some early reports suggested that HCC recurrence rates may be greater after LDLT than DDLT, but overall survival rates were similar or superior to DDLT [83,84]. However, more recent studies have not shown a statistical difference in overall or recurrence-free survival [85,86]. In the LDLT setting, recurrence may be linked to low graft-to-recipient weight ratio [87] in addition to tumor biology. Over time, the limits of acceptable tumor burden for LDLT have increased over time as centers have improved at assessing recurrence risk [86,88,89].

LDLT is also increasingly utilized for oncologic indications outside HCC. Patient survival rates after LDLT for pCCA can reach 66.5% at 5 years, although the advanced biliary reconstruction that is often required may lead to additional complications [90]. Recently, case reports and small case series have also been published detailing LDLT for iCCA [91,92,93]. Reports on LDLT for mixed HCC-CCA have been mixed, with some finding similar survival rates to HCC [94] and others finding reduced survival [95]. LDLT for CRLM is increasingly well-studied, with multiple case series reporting 3-year post-transplant overall survival rates upwards of 93.8% [96,97]. Promising results have also been published on patients with NET metastases [98].

### 4.2. Extended Criteria Donors

Extended criteria donors (ECDs), also referred to as marginal donors, have been utilized to expand the deceased donor pool. Although there is no single universally accepted definition of ECDs, most centers would include donation after circulatory death (DCD), advanced-age donors, steatotic grafts, split liver grafts, and grafts with prolonged graft ischemia time [99,100,101,102]. These donors have traditionally been classified as high risk, but because they may be less preferred than standard criteria donors, wait times for extended criteria organs can be shorter in some circumstances. Studies have shown that a majority of patients are willing to accept these organs when properly informed of the risks of such grafts [103].

It has been suggested that patients with HCC may be good candidates for accepting ECD grafts because most have low physiologic MELD scores [101,104]. This approach allows improved access to transplantation for patients with HCC who are not eligible for exception points [105]. Transplant centers have increased their utilization of ECD grafts over time [106]. Recent analysis of US SRTR data showed a small but statistically significant reduction in OS after LT for HCC when utilizing either donation after brain death ECD grafts or DCD grafts [107]. However, the survival difference of <3% at 5 years post-LT is dwarfed by the survival benefit of undergoing LT, which may not have occurred if the patient did not receive an ECD liver.

### 4.3. Machine Perfusion

The recent adoption of machine perfusion (MP) in LT has early favorable outcomes in select settings of transplantation for cancer. Normothermic machine perfusion (NMP), introduced in 2012, preserves donor livers at body temperature using oxygenated, nutrient-rich perfusate delivered through dual perfusion of the portal vein and hepatic artery [108]. This approach supports active cellular metabolism and allows continuous monitoring via oxygenation sensors. Key indicators such as bile production, perfusate lactate levels, factor V synthesis, glucose metabolism, galactose clearance, and lower hepatocellular enzyme levels in the perfusate help determine graft function prior to transplantation [109]. Randomized controlled trials utilizing NMP for donor organs have demonstrated reduction in both transaminases and early allograft dysfunction rates in recipients [110,111,112]. In addition, some studies have suggested that MP may decrease the risk of recurrence, likely by ameliorating the effects of ischemia–reperfusion injury, which has been linked to higher HCC recurrence post-LT [113,114].

Hypothermic oxygenated perfusion (HOPE) requires only 1–2 h of perfusion before implantation, though perfusion time can be extended to cover the entire preservation period [115]. HOPE has demonstrated benefit in post-transplant outcomes for HCC including reduced risk of ischemia- reperfusion injury, cholangiopathy, recurrence rates in HCC and increased 5-year recurrence free survival compared to static cold storage [116,117].

Tang et al. demonstrated a benefit of normothermic machine perfusion on recurrence free survival at 1- and 3-years post-transplant compared to static cold storage, although overall survival was not significantly different [118]. Although early retrospective studies show promise and a benefit of machine perfusion in liver transplant for liver cancer, larger randomized trials are needed with standardized protocols and cost analyses across broader oncologic indications.

### 4.4. Access, Outreach, and Education

LT provides superior oncologic outcomes for transplant-eligible patients with liver cancer, but unfortunately, not all patients have equal access to transplantation. Racial and ethnic minorities are less likely to be placed on the LT waitlist and to undergo LT, as are patients from socioeconomically disadvantaged backgrounds [119,120,121,122,123,124,125,126]. Poor health literacy among disadvantaged individuals can also lead to poor post-transplant outcomes [123]. Additionally, patients with lower education levels or public insurance are less likely to receive LDLT [127]. Historically, women were also less likely to undergo LT than men, likely due to the shorter stature of many women and more frequent donor–recipient size mismatches [123]. This discrepancy has led to changes in organ allocation and the new MELD 3.0 allocation system, where women receive higher MELD scores to alleviate these differences. Unfortunately, despite attempts to address known disparities, there is a very real problem with access to transplantation among persons from disadvantaged communities and backgrounds.

These disparities also exist in transplant oncology—studies using US national data sets have shown Black patients with HCC are less likely to undergo LT [128,129]. They also experience higher mortality after LT for HCC than White patients, with Asian and Hispanic patients having improved survival relative to White patients [129,130]. Female patients are also less likely to receive LT for HCC, particularly if they have alcohol-associated liver disease [131]. Additionally, patients with public insurance or who are from low-income census tracts are less likely to be referred for LT for HCC [132]. It is also important to note that many insurance plans do not cover LT for less common liver cancers, such as iCCA, including Medicare. Thus, there is a very real population of patients with liver cancer who would benefit from LT but who are unable to pay for the procedure. However, there is some evidence that these disparities have improved over time [133], highlighting the need to increase efforts to increase awareness and interventions that address barriers in access to LT for oncologic indications [134].

In addition to education and outreach, community engagement to promote health equity, additional changes like implementing implicit bias training for healthcare providers, allocation models to address geographic inequities and additional policy changes will be necessary to combat these discrepancies. These changes can promote equitable referral and evaluation practices, ensuring fairer treatment for all transplant candidates [135].

## 5. Conclusions and Future Directions

Transplant oncology is a rapidly evolving field and has been the center of many advancements in the field of transplantation. Because LT generally provides superior outcomes to resection or non-surgical therapies, new developments in this field have the potential to improve outcomes for many types of liver cancer. Oncologic indications for LT have grown over the past several decades, allowing patients with larger disease burden and non-HCC primary and secondary liver cancer to receive this life-saving treatment. Although the expansion of indications and eligibility for MELD exception points has increased the number of HCC patients eligible for LT, research has shown that patients with even greater tumor burden also experience excellent post-LT survival if afforded the chance to undergo transplantation. To allow for greater access to LT for primary and secondary liver cancer, the liver donor pool must also increase. Strategies such as increased utilization of extended criteria donors, with or without machine perfusion or other organ-preservation techniques, and LDLT will help bridge the donor–candidate gap. Advancements in systemic and locoregional therapies for liver cancer may allow more patients to be adequately downstaged or bridged to transplant. The field will also benefit from efforts to address disparities in access to transplant due to social determinants of health and other socioeconomic factors. Combined, these strategies show great promise toward extending the lives of many patients with liver cancer.

## Figures and Tables

**Table 1:** United States United Network for Organ Sharing (UNOS) liver graft allocation exception points guidelines for patients with primary or secondary liver cancer.

Cancer	MELD Exception Eligibility	Downstaging Eligibility	Wait Period
Hepatocellular Carcinoma	T2 stage HCC: 1 lesion > 2 cm and <5 cm, or 2–3 lesions > 1 cm and <3 cm (LI-RADS 5 or biopsy-proven)	Patients beyond T2 who are successfully downstaged to T2 may qualify, provided: No extrahepatic metastasis No macrovascular invasion AFP ≤ 1000 ng/mL	6 month wait time MMaT-3
Hilar Cholangiocarcinoma	Malignant-appearing stricture and at least one of the following: Positive biopsy or cytology CA 19-9 > 100 U/mL (in absence of cholangitis) Aneuploidy	If a mass is present, it must be single and <3 cm in radial diameter (measured perpendicular to the bile duct). Longitudinal extension is not considered	No wait time MMaT-3
Intrahepatic Cholangiocarcinoma	Single iCCA lesion ≤ 3 cm in diameter, confirmed by imaging or biopsy in patients with cirrhosis	Unresectable No evidence of metastasis	6 month wait time MMaT-3
Neuroendocrine Tumors	Must be confined to the liver, bi-lobar, and not amenable to resection.	Primary resected and No evidence of recurrence for at least 6 months prior to MELD exception request.	6 month wait time MMaT-3
Colorectal Liver Metastases	Histological diagnosis of colon/rectal adenocarcinoma At least 12 months from time of CRLM diagnosis to time of initial exception request	Primary resected No extrahepatic disease	12 month wait time MMaT-20

**Table 2:** Five-year post-liver transplant survival outcomes for patients with primary or secondary liver cancer.

Malignancy	Indication	Survival	References
Hepatocellular carcinoma	Cirrhosis with HCC within Milan/UCSF criteria	5-year OS 70%	[8]
Colorectal liver metastases	Unresectable liver-only disease, low Oslo score, stable post-chemotherapy	5-year OS ~60–83% (SECA I/II, TRANSMET)	[15,16,17]
Intrahepatic cholangiocarcinoma	Small tumors with favorable biology (<2 cm)	5-year OS ~50–60% in selected patients	[18]
Hilar cholangiocarcinoma	Unresectable tumors with neoadjuvant chemoradiation and strict selection	5-year OS ~65% (Mayo protocol)	[19]
Neuroendocrine tumor metastases	Liver-dominant, low-grade tumors, stable disease > 6 months	5-year OS ~70–90%	[20]
